# Urine Metabolite Profiling of Human Colorectal Cancer by Capillary Electrophoresis Mass Spectrometry Based on MRB

**DOI:** 10.1155/2012/125890

**Published:** 2012-12-02

**Authors:** Jin-Lian Chen, Jing Fan, Liu-Shui Yan, Hui-Qin Guo, Jing-Jing Xiong, Yan Ren, Jun-Duo Hu

**Affiliations:** ^1^Department of Gastroenterology, Shanghai East Hospital, Tongji University School of Medicine, Shanghai 200120, China; ^2^Department of Gastroenterology, Shanghai Sixth People's Hospital, Shanghai Jiaotong University, Shanghai 200233, China; ^3^Medical College, Soochow University, Suzhou Jiangsu, 215213, China; ^4^Cyclization College, Nanchang Aeronautical University, Jiangxi, Nanchang 330063, China

## Abstract

*Aim*. The study was to investigate the metabolic profile of urine metabolites and to elucidate their clinical significance in patients with colorectal cancer. *Methods*. Colorectal cancers from early stage and advanced stage were used in this study. Urine samples of colorectal cancer patients and healthy adults were collected and subjected to capillary electrophoresis mass spectrometry based on moving reaction boundary analysis. The metabolic data were analyzed by SPSS 17.0 to find urinary biomarkers for colorectal cancer. *Results*. The results indicated that the urine metabolic profiling of colorectal cancer patients had significant changes compared with the normal controls, and there were also differences between early stage and advanced colorectal cancer patients. Compared with the control group, the levels of isoleucine, valine, arginine, lactate acid and leucine increased (*P* < 0.05), but those of histidine, methionine, serine, aspartic acid, citric acid, succinate, and malic acid decreased in urine samples from colorectal cancer (*P* < 0.05). Furthermore, the levels of isoleucine and valine were lower in urine of patients with advanced colorectal cancer than those in early stage colorectal cancer (*P* < 0.05). *Conclusion*. The technique of capillary electrophoresis mass spectrometry based on MRB could reveal the significant metabolic alterations during progression of colorectal cancer, and the method is feasible and may be useful for the early diagnosis of colorectal cancer.

## 1. Introduction

Colorectal cancer (CRC) is one of the most common malignancies and a leading cause of cancer-associated death worldwide, especially in Europe and the United States [1–3]. Recently, the incidence rate of colorectal cancer has been increasing with lifestyle changes [4]. Though colorectal cancer is so common, we do not have effective treatment to cure it. Until now, the only curative treatment option for it is surgical resection [5]. However, the 5-year survival rates remain low, only 8% for stage IV patients, but are 93% for stage I patients [6]. So it is of significance to make a prediction about the oncogenesis and metastasis at the early stage of colorectal cancer in order to improve the prognosis. But we do not have effective measures to diagnose colorectal cancer at the time when the disease occurred as soon as possible for they have no specific clinical symptoms until in the late stage. To date, the most effective screening method for precancerous lesions and cancer morbidity in colon (e.g., aberrant crypt foci, polyps, and tumors) is colonoscopy [7]. However, the results of endoscopy are significantly affected by artificial factors (e.g., the experience of the gastrointestinal pathologist) [8, 9]; moreover, colonoscopy is invasive, and the procedure is unpleasant [2]. Although fecal occult blood testing (FOBT) and some certain tumor biomarkers, such as carcinoembryonic antigen (CEA), have been used in clinical dignosis, the sensitivity and specificity are relatively poor [10, 11]. Therefore, many researchers are trying to find novel biomarkers for the early detection of colorectal cancer.

In our study, we attempted to discover new biomarkers using a metabolomics approach for colorectal cancer. Metabolomics is the rapid development of new research areas following the genomics, transcriptome, and proteomics [12], the analytical method to study low molecular weight compounds and of potential for development in system biology [4, 13], which can be used to analyze the changes in metabolite levels in biological samples [14]. Though functional genomics methods such as transcriptome and proteomics can simultaneously detect expression changes of a large number of genes or proteins under the influence of drugs, disease, environmental, or other factors, these changes could not establish direct relation with the changes of the biological functions. Metabolomics is the endpoint of the omics cascade in other words the last step in the cascade before the phenotype [4], and therefore it can establish a direct correlation for changes between metabolite levels and biological phenotype changes. At present, metabolomic methods include nuclear magnetic resonance spectroscopy (NMR), liquid chromatography-mass spectrometry (LC-MS), gas chromatography-mass spectrometry (GC-MS) and capillary electrophoresis mass spectrometry (CE-MS) [15]. NMR requires a large number of samples as a result of the relatively poor sensitivity [12]; LC-MS needs derivation, which is a tedious complex process and is complicated to operate; GC-MS is of high sensitivity, but mainly for the analysis of volatile and semivolatile metabolites, and there are still some challenges for the nonvolatile metabolites [16]. Capillary electrophoresis mass spectrometry can overcome the shortcomings of the above methods, which not only has excellent resolution capability but also requires only trace amounts of sample. The main advantages of coupling capillary electrophoresis with mass spectrometry are the high resolution, good separation speed, sensitivity, and selectivity, which can detect almost any kind of charged compound [17]. Nevertheless, the detection sensitivity is limited due to the less injection of capillary electrophoresis. Our recent study has shown that moving reaction boundary (MRB) could stack analyte in capillary electrophoresis to improve the detection sensitivity [18]. In this study, we apply the technology of capillary electrophoresis mass spectrometry based on moving reaction boundary to achieve online enrichment and qualitative and quantitative detection of metabolites in the urine of patients with colorectal cancer.

## 2. Materials and Methods

### 2.1. Chemicals

Twenty kinds of amino acid solution and nine kinds of organic acid solution were purchased from Sigma (Sigma-Aldrich, Germany). HPLC-grade methanol (MeOH) was from Fisher Company. Formic acid (FA) (A.R) was supplied by Beijing Chemical Factory. Ammonium formate (A.R) was provided by Beijing Chemical Factory. Sodium hydroxide was purchased from West Long Chemical Co., Ltd., and Ammonia was from Hongdu Biochemistry Co., Ltd. (Jiangxi province, China). Standard solution was prepared with water from Milli-Q water purification system (Millipore).

### 2.2. Patients and Urine Samples

Urine samples were collected from 20 CRC patients (aged 37 to 87 years old) and 14 healthy volunteers (female/male, 6/8, aged 50 to 86 years old, and the median age was 68 years old) from Shanghai Sixth People's Hospital, Shanghai Jiaotong University (Shanghai, China). All the CRC patients were diagnosed by colonoscopy combined with pathological examination and stages according to the seventh edition of the International Union Against Cancer (UICC) TNM: stage I and II (early stage cancer), 8 patients (female/male, 5/3), aged 37 to 87 years (the median age was 77 years old); stage III and IV (advanced stage cancer), 12 patients (female/male, 5/7), aged 39 to 80 years (the median age was 69 years old). All subjects signed an informed consent under local research ethics committee approval. We collected the urine samples with centrifuge tubes of 5 mL temporarily early in the morning between 6.00 and 7.00 AM, centrifuged immediately; then the supernatants were transferred to frozen tubes and stored at −80°C until processing. All patients had not received chemotherapy or radiotherapy treatment before sample collection. Prior to CE-MS analysis, the urine samples were removed from the refrigerator to thaw at room temperature, transferred to centrifuge tubes of 1.5 mL, and centrifuged (12 000 rpm) for 10 min; subsequently the supernate was filtered through a 0.22 *μ*m organic membrane. Mix the sample with sample buffer with the volume ratio of 1 : 1 before injection of samples into the capillary.

### 2.3. Instrumentation and Procedures

For CE-ESI-MS experiments, the HP^3D^CE capillary electrophoresis instrument (Agilent, USA) was coupled to a 1100 series MSD TRAP (VL) mass spectrometer (Agilent, USA) applying a custom ESI sheath-flow interface with nebulizer gas [19]. Uncoated fused silica capillaries were from Ruifeng Chromatography Devices Co., Ltd. (Yongnian, Hebei, China) with a total length of 90 cm and an inner internal diameter of 50 *μ*m. Formic acid (1.2 M, pH 1.8) was used as background electrolyte (BGE), while ammonium formate (60 mM, pH 10.2) was as the sample buffer. Sample injection was performed hydrodynamically for 80 s at 50 mbar. The volume of the sample plug injected corresponds to 61 nL. We rinsed the new dare fused-silica capillaries with 1 M sodium hydroxide for 30 min at 930 mbar, followed by deionized water for 20 min at 930 mbar, then 0.1 M sodium hydroxide for 20 min at 930 mbar, and followed by deionized water for 20 min at 930 mbar. At the start of the day, capillaries were flushed with deionized water for 15 min at 930 mbar and with 0.1 M sodium hydroxide for 15 min at 930 mbar and then with deionized water for 15 min at 930 mbar. Between the runs, we rinsed capillaries with deionized water for 2 min, 0.1 M sodium hydroxide for 3 min, deionized water for 2 min, and background buffer for 3 min in succession. During the rinsing step, the end plate voltage, the nebulizer capillary voltage, and capillary voltage were set to 0, which prevented these washing solutions from getting into the vacuum part of the mass. We set the capillary temperature at 25°C and performed the separation voltage at +30 kV. Spectra were collected with a time resolution of 0.5 s. Masses were obtained from 50–350 m/z. CE-MS coupling was realized by a coaxial sheath liquid interface (Agilent, USA) with methanol-water-formic acid (50 : 50 : 0.1, v/v/v) as sheath liquid. The following spray conditions were used: ESI in positive ionization mode voltage was 4.5 kV; sheath liquid flow, 9 *μ*L/min; dry gas temperature, 275°C; nitrogen flow, 10 L/min; nebulizer pressure, 0.5 bar.

### 2.4. Analytical Validation

Linearity of response for metabolites was evaluated by standard measurement of six concentrations ranging from 10 to 200 *μ*M (10, 20, 50, 100, 150, and 200 *μ*M). Linearity of response for the metabolites was also measured in the same concentration range in pooled human urine. The reliability of the analysis of CE/MS detection was verified by 5 endogenous urinary metabolites (L-arginine, L-histidine, L-aspartic acid, lactic acid, and citric acid) of representative. The standards of these metabolites were added into the blank urine in accordance with low (20 *μ*M), middle (50 *μ*M), and high (80 *μ*M) concentration; each quality control (QC) sample was repeatedly measured three times continuously.

### 2.5. Data Analysis

CE-MS data were analyzed using principal component analysis (PCA) to differentiate the samples. Each sample was represented by a CE/MS TIC. The absolute peak area of each compound was calculated as the response after the peak areas of compound were integrated. *t*-test was applied to compare the differences between two groups. For three groups or more, one-way ANOVA test was performed. Data were expressed as mean ± SD. The differentially expressed compounds were considered statistically significant with *P*  value < 0.05. Statistical analyses were carried out using SPSS 17.0 for windows.

## 3. Results

### 3.1. Detection of the Standards

**Figure 1:**
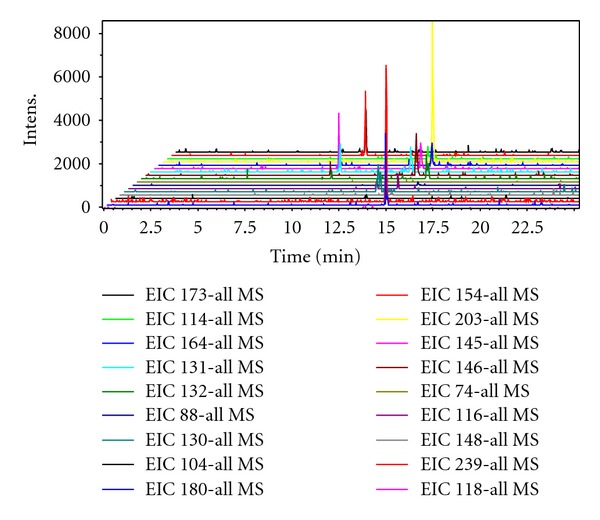
Total ion chromatogram of 18 kinds of amino acid standards.

**Figure 2:**
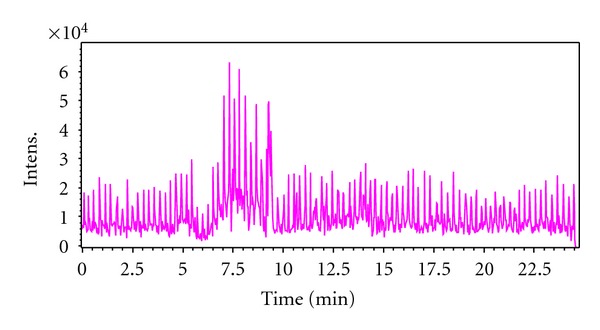
Total ion chromatogram of the urine of a patient with colorectal cancer.

The chromatogram of 18 kinds of amino acid standards was shown in Figure 1. From the figure, we can see that 16 kinds of amino acids were separated effectively under such conditions, and the peak times were concentrated in 10–17 minutes. Substances in the actual urine samples also showed good separation from the chromatogram in Figure 2.

### 3.2. Recoveries and Precision Results

The standards of metabolites (L-arginine, L-histidine, L-aspartic acid, lactic acid, and citric acid) were added into the blank urine in accordance with low (20 *μ*M), middle (50 *μ*M), and high (80 *μ*M) concentration; each QC sample was repeatedly measured three times continuously. The result is showed in Table 1.

### 3.3. Evaluation of CE-ESI-MS Method for Metabolic Profiling

Recently, a CE-ESI-MS method has been developed for the highly efficient and sensitive analysis of metabolites (amino acids and organic acids) in human urine [19]. Here we demonstrate that MRB-CE-ESI-MS method can be used for the profiling of amino acids and organic acids. In CE, amino acids and organic acids (ionogenic compounds with electrophoretic properties) migrate toward the cathode along with the electroosmotic flow which is the MS in our setup. To analyze amino acids and organic acids simultaneously, 1.2 M formic acid with low pH was used to confer positive charges on these ionic compounds, making them amenable to MS analysis. Figure 1 shows chromatogram of 18 kinds of amino acid standards; 16 amino acids were separated in less than 18 min. Figure 2 displays a total ion electropherogram of urine from a colorectal cancer patient obtained by CE-ESI-MS analysis; around 100 signals were detected in the sample. The analytical quality of the data obtained from the CE-ESI-MS analysis of QC samples was stated in Table 1. The five QC samples before and after addition of known analyte concentrations (20, 50, and 80 *μ*M) were analyzed as shown in Table 1. Recoveries ranged from 71.83% to 88.70%, and the RSDs were 1.92%–6.70% for all analytes.

### 3.4. Metabolic Profiling and Multivariate Statistics

We used the CE-ESI-MS method for the metabolic profiling of urine samples from 20 patients with CRC and from 14 control subjects. All data was obtained by LC/MSD Trap Software Version 5.2 based on internal standard; most of the total ion chromatograms were identified as endogenous organic acids and amino acids involved in several metabolic pathways, such as glycolysis (lactic acid), serine metabolism (serine), and tricarboxylic acid (TCA) cycle (succinate, citric acid, and malic acid).

The CE-ESI-MS data about urine metabolites in CRC and normal control subjects were analyzed by *t*-test after normalization of data. The marker metabolites selected by *t*-test are displayed in Table 2. Among the metabolites, the lactic acid, arginine, isoleucine, leucine, and valine were significantly upregulated, while the citric acid, histidine, methionine, serine, aspartate, malic acid, and succinate were remarkably downregulated in CRC compared to the normal control subjects (*P* < 0.01). The main metabolic pathways related to CRC included glycolysis (lactic acid), serine metabolism (serine), tricarboxylic acid (TCA) cycle (succinate, citric acid, and malic acid). The levels of valine and isoleucine were lower in advanced stage colorectal cancer group than the early colorectal cancer group as shown in Table 3, *P* < 0.01.

**Figure 3:**
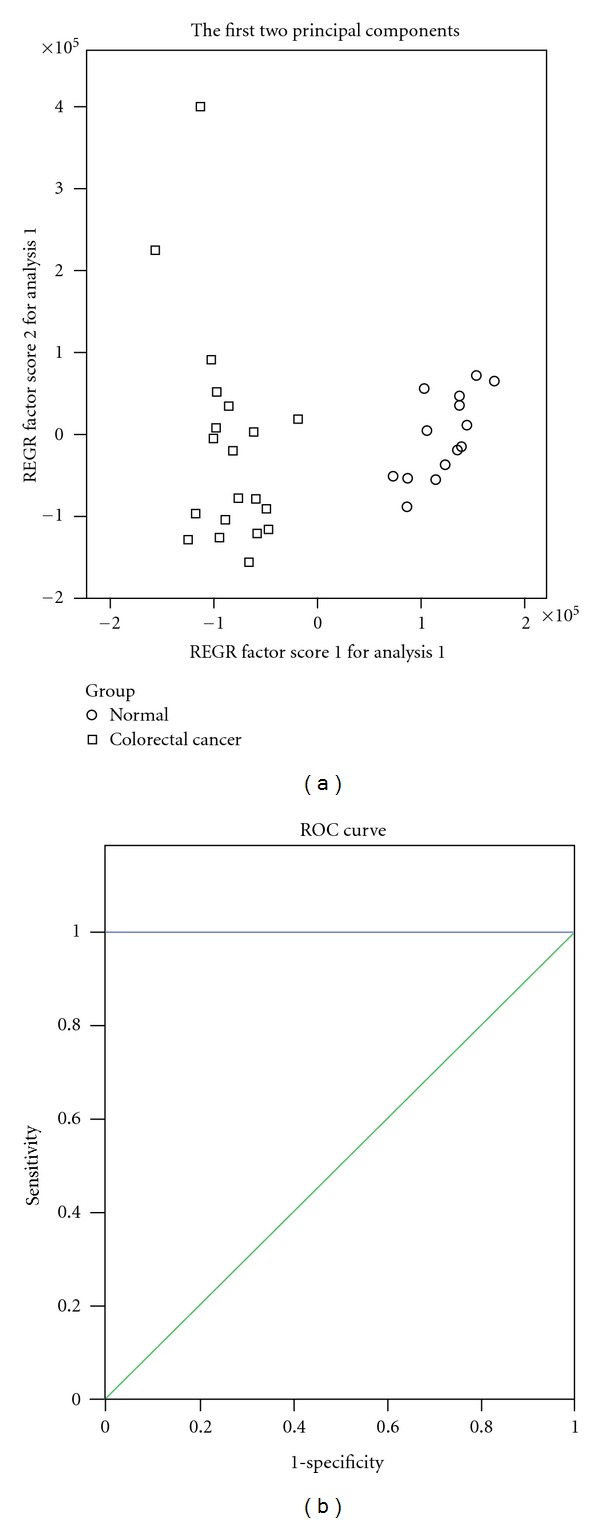
Principal component analysis model and receiver operating characteristic curve for colorectal cancer. (a) Principal component analysis (PCA) scores plot of colorectal cancer urine specimens from control specimens based on 12 marker metabolites. The PCA scores plot showed that different samples (normal group, colorectal cancer group including early stage group and advanced group) were scattered into different regions; (b) receiver operating characteristic (ROC) analysis was performed using the values determined by the first two principal components. Area under the curve (AUC) = 1.00.

**Figure 4:**
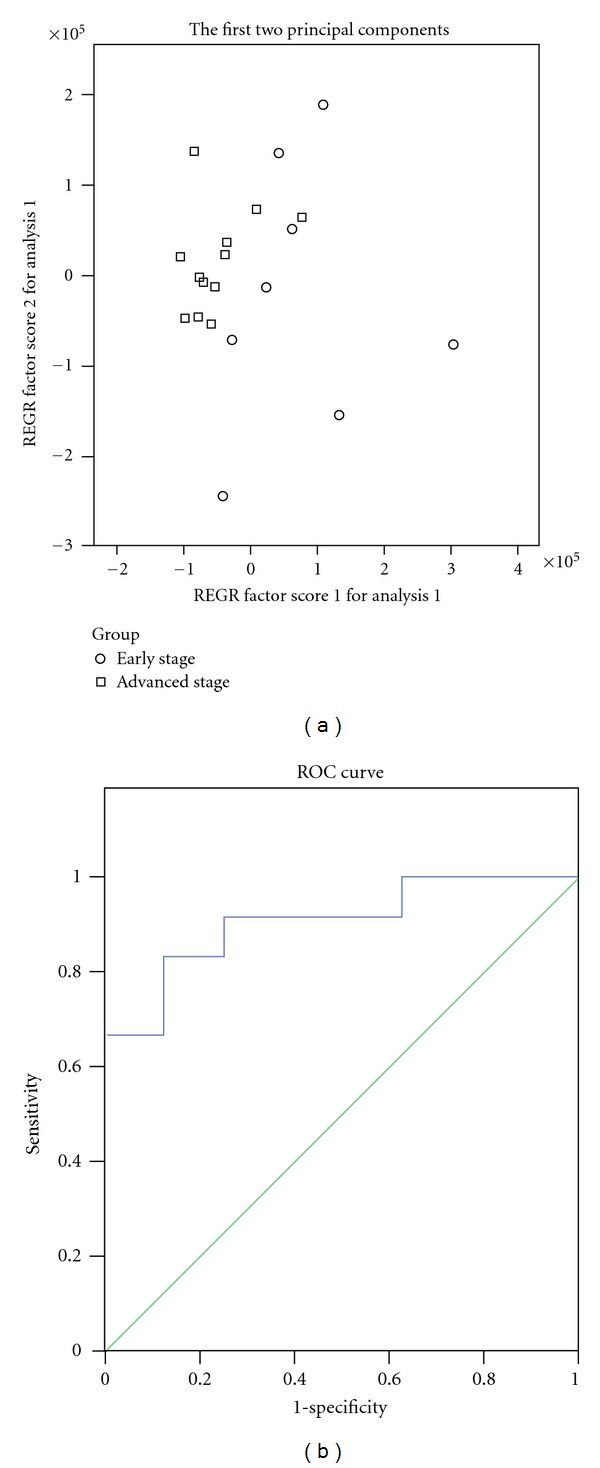
Principal component analysis model and receiver operating characteristic curve for advanced colorectal cancer. (a) Principal component analysis (PCA) scores plot of early stage group and advanced group based on 2 marker metabolites. The PCA scores plot showed that the samples from early stage group and advanced group were scattered into two different regions; (b) receiver operating characteristic (ROC) analysis was performed using the values determined by the first two principal components. Area under the curve (AUC) = 0.906.

A PCA model for colorectal cancer was constructed taking the marker metabolite areas as variables (lactic acid, arginine, isoleucine, leucine, valine, citric acid, histidine, methionine, serine, aspartate, malic acid, and succinate). The PCA scores plot illustrated that the CRC group and the normal control group were scattered into different regions (Figure 3(a)). ROC analysis was performed using the values of the first two principal components of the PCA model, which confirmed the reliability of the PCA. The specificity and sensitivity trade-offs were summarized for each variable with AUC. The AUC value of the PCA model is 1.00 (Figure 3(b)), which verified a good diagnostic value for colorectal cancer. Furthermore, another PCA model for advanced stage of CRC constructed by two metabolites could differentiate between the early stage group and the advanced group (Figure 4(a)). The PCA model was confirmed by the receiver operating characteristic (ROC) analysis with AUC = 0.906 (Figure 4(b)).

## 4. Discussion

Colorectal cancer is the second and fourth most cause of cancer-associated death in Europe and the USA, respectively [3]. In Singapore, colorectal cancer is the first most common cancer in males and the second in females; also CRC is the first most common cancer in Singapore when both genders are put together [20]. Recently, the number of colorectal cancer patients has been increasing because of the changes of lifestyle [4]. The main method to improve the prognosis of patients with colorectal cancer is early diagnosis and early treatment. Fecal occult blood test (FOBT) is an effective screening tool to colorectal cancer but the sensitivity is poor [4, 21]. Though the endoscopic examination of colon is the gold standard for diagnosis, it is invasive and unsuited to decrease the risk of morbidity and mortality [4]. The approaches used in colorectal cancer staging and prognosis assessment have many limitations [22]. Capillary electrophoresis mass spectrometry has been developed rapidly because of its many superiorities in detecting metabolites of urine and other body fluids, such as the high resolution power, good separation speed, and sensitivity and selectivity [17]. Despite this, the detection sensitivity is limited for the less injection of capillary electrophoresis. Moving reaction boundary (MRB) can stack analytes in capillary electrophoresis to improve the detection sensitivity [18]. In this method, a neutralization reaction is formed between the background buffer (1.2 M formic acid pH = 1.8) and the sample buffer (60 mM ammonium pH = 10.2), when an electrical field is imposed. The neutralization leads to the formation of a progressive low-conductivity zone in the original matrix plug, and then the low-conductivity zone further results in pH-mediated-induced field-amplified sample injection (FASI) stacking [23]. Moving reaction boundary was formed by the electromigration reaction which can be used to enrich the samples. The mechanism of preconcentration is as follows: the capillary is filled with background buffer of high conductivity, and the sample buffer is injected into the capillary to a certain length subsequently. After the sample is injected completely, a high positive voltage is applied, the greater electric field causes the ions to migrate more rapidly across the sample zone. Once the ionic analytes arrive at the boundary between the sample zone and the background buffer, the electric field strength suddenly weakened and migration becomes slower, causing the sample analytes to be concentrated near the boundary. For the mobility of electroosmotic flow (EOF) is rapider than those of the charged analytes, all the charged analytes will finally move toward the mass spectrometry side, while the analytes in samples are separated by the CE mode [24]. To our knowledge, this is the first metabolomic investigation of urine with colorectal cancer using capillary electrophoresis mass spectrometry based on MRB.

In our study, twelve differential metabolites were found between colorectal cancer and normal control subjects, while two differential metabolites were identified between early stage and advanced colorectal cancer. PCA analysis showed there were significant differences among normal controls and colorectal cancer patients, including 8 patients with early colorectal cancer.

The lactic acid level was found to be higher in CRC urine. Lactic acid is the end product of glycolysis that increases rapidly during the occurrence and development of tumor. Cancer cells are in a high metabolic state, and the rate of aerobic glycolysis becomes higher, with more glucose converted into lactic acid even in the presence of oxygen, which is known as the “Warburg effect” [9, 20, 25]. The increased lactate level has been demonstrated in various tumors, including renal, head and neck, and gastric cancers [9, 26–28]. We found the lower levels of citric acid, malic acid, and succinate in the CRC urine specimens, which are most likely related to the deregulation of the tricarboxylic acid cycle and the increased demand for energy in tumors [20, 28]. The isoleucine is ketogenic amino acid which decomposed into acetyl-coenzyme A and succinatethe-coenzyme A, the important substances of the tricarboxylic acid cycle, which were greatly required during the gluconeogenesis [29], so the isoleucine level is higher in the cancer group compared to the normal group. The higher level of valine in CRC group than normal group is also related to the increased glycolysis in tumor cells [30]. Aspartic acid is one of the essential substances of the *in vivo* synthesis of nucleotides, which is needed in large amounts for the synthesis of nucleotides to promote tumor development when the tumor cells proliferate; therefore, the content of aspartic acid in the body is expressed at lower level than normal control group. Serine was decreased in colorectal cancer patients compared to the controls because serine takes part in glycolysis or tricarboxylic acid cycle to provide more energy for the progression of tumor [31]. Arginine is known to be related to immune function [32]. It has been reported that arginine can inhibit chemical-induced colorectal cancer and reduce cell proliferation in colorectal adenoma patients [33, 34]. We found histidine was downregulated in CRC patients compared with normal control subjects. The mechanism may be the higher activity of histidine decarboxylase leading to the accelerated decarboxylation of histidine to histamine [2]. Methylation is one of the mechanisms for regulating cell growth and differentiation; S-adenosyl-L-methionine is a methyl donor which is generated by the conversion of methionine; therefore the level of methionine was down-regulated during the process of tumor occurrence and development [12].

In our study, we also found that the levels of valine and isoleucine were lower in the advanced colorectal cancer group compared with the early stage group. It could be explained by the hypothesis of Yamanaka and the workers [29]; obstacles produced by increasing muscle tissue protein improved the intrahepatic glucose gluconeogenesis and added the oxidation of BCAA in muscle, which is the main mechanism of the elevated levels of BCAA for patients with early colorectal cancer, but the further development of tumor can stop the increasing barriers, and therefore the BCAA levels may decline.

## 5. Conclusion

To the best our knowledge, this is the first report to evaluate the variations of urine metabolites among colorectal cancers using capillary electrophoresis mass spectrometry based on MRB. In our study, we filter out 12 marker metabolites between normal control subjects and colorectal patients and 2 marker amino acids between early stage and advanced colorectal cancers. The results suggest that the technique of capillary electrophoresis mass spectrometry based on MRB is feasible and may be useful for the diagnosis of colorectal cancer.

## Figures and Tables

**Table 1:** Recoveries and precision of QC samples (*n* = 3).

Metabolite	Adding amount (*μ*M)	Detection value (*μ*M)	Average recovery (%)	RSD (%)
Arginine	20.0	16.0, 17.0, 18.0	85.00	5.88
	50.0	43.0, 44.0, 46.0	88.70	3.45
	80.0	68.0, 71.0, 73.0	83.33	2.29
Histidine	20.0	14.0, 15.0, 16.0	75.00	6.70
	50.0	43.0, 44.0, 45.0	88.00	2.27
	80.0	62.0, 65.0, 67.0	80.80	3.89
Aspartate	20.0	14.0, 15.0, 15.0	73.33	3.94
	50.0	39.0, 41.0, 42.0	81.33	3.76
	80.0	63.0, 65.0, 68.0	80.83	2.36
Lactic acid	20.0	15.5, 16.0, 17.3	81.33	5.71
	50.0	42.6, 44.2, 43.0	86.53	1.92
	80.0	68.5, 72.4, 70.2	87.96	2.78
Citric acid	20.0	13.6, 14.5, 15.0	71.83	4.94
	50.0	39.4, 41.6, 43.0	82.67	4.39
	80.0	68.3, 70.5, 74.0	88.67	4.05

**Table 2:** Different metabolites identified in normal control group and colorectal cancer group (mean ± SD).

Metabolites	Normal control group (*n* = 14)	Colorectal cancer group (*n* = 20)	*P*
Leucine	497444.00 ± 108999.397	1074016.00 ± 456521.184	0.000
Isoleucine	612788.64 ± 93219.817	1134260.20 ± 496322.174	0.000
Valine	538171.79 ± 100157.421	995357.85 ± 347273.028	0.000
Arginine	456031.93 ± 117404.005	1179011.50 ± 548364.933	0.000
Histidine	1168892.57 ± 317549.609	649361.35 ± 168817.630	0.000
Methionine	1261904.93 ± 468921.459	713061.95 ± 189689.901	0.000
Aspartate	1896296.43 ± 921667.854	681982.95 ± 122738.857	0.000
Serine	1023781.50 ± 203670.927	657044.45 ± 123487.631	0.000
Lactic acid	619754.64 ± 129620.274	1168716.15 ± 339239.261	0.000
Succinate	965363.21 ± 161307.936	611380.10 ± 144727.123	0.000
Citric acid	1298679.71 ± 610999.062	642247.10 ± 224070.728	0.002
Malic acid	1083173.43 ± 203204.078	613131.90 ± 164751.873	0.000

**Table 3:** Different metabolites identified in early stage group and advanced group (mean ± SD).

Metabolites	Early stage group (*n* = 8)	Advanced group (*n* = 12)	*P*
Valine	1315695.50 ± 337981.724	781799.42 ± 105973.802	0.003
Isoleucine	1584647.00 ± 463637.420	834002.33 ± 206929.304	0.000
